# Hospital and Institutional News

**Published:** 1917-05-05

**Authors:** 


### HOSPITAL AND INSTITUTIONAL NEWS.

### KING MANOEL INSPECTS A LIVERPOOL WAR INSTITUTION.

As the representative of the orthopædic section of the British Red Cross Society, King Manoel paid a visit to Liverpool one day last week for the purpose of inspecting the curative workshops connected with the Alder Hey Military Hospital, where the results achieved up to the present have been of a most encouraging character. Escorted by Colonel Robert Jones, C.B., Inspector-General of Orthopædics to the British Army, and Captain Broad, the medical head of the curative workshops, King Manoel watched wounded soldiers engaged in the rudiments of the handicraft which will provide them in the future with a livelihood, and will also do much to restore the use of muscles and limbs impaired in the fighting.

### A FRIEND AT COURT.

A sum of £2,000 which, from a bequest recently entrusted to him by the late Mrs. Wheeley Lea, the Bishop of Worcester at his discretion has allotted to the Worcester General Infirmary, should remind the casual how valuable is the support which the clergy can and do give to hospitals. The Bishop of Worcester has taken a personal interest in the institution, but every clergyman is a friend at court for the voluntary hospital. The clergy are invaluable links between the public and the hospitals, apart from their services as chaplains—services, by the way, which are often underpaid. At Worcester there has been some want of common interest between the hospitals and the public, the proof of which has been the constant financial difficulty with which the General Infirmary has been beset. We welcome the recent bequest (half of which comes as a legacy, and half as an investment yielding a perpetual annual subscription). It is a proof not only that a Bishop's discretion may be trusted, but that the tide has turned in the right direction.

### CHARGING HOSPITAL PATIENTS FOR DRUGS.

A complaint has been made against the Queen's Hospital, Birmingham, which is worth notice, because the answer which it has elicited is an indication of the difficulties of the times. A patient, in applying for medicine, was invited to pay a small charge, and consequently wrote a letter of protest to the local newspapers. A representative of the Hospital Saturday Fund has taken the matter up, and, by way of reply, states that the high prices of drugs, food, and coal, led to a decision to discontinue the free supply of medicine except in necessitous cases. He therefore suggests that the patient would have been more considerate if he had informed the superintendent of his circumstances (he is an old-age pensioner), when the medicine would probably have been given to him. The Queen's Hospital, Birmingham is understood to be not the only hospital which has adopted this course, but we do not regret the complaint, since it reminds the public of the difficulties which hospitals and their patients have to face at the present time.

### IMPORTANT TO RESIDENTS IN LONDON.

Residents in the districts under the jurisdiction of several of the Borough Councils within the Metropolitan area, and especially in those of Paddington and St. Pancras, have experienced callous neglect on the part of the Borough Council for periods of weeks during the past winter, which amounted to a grave scandal. Judging by the reception accorded to remonstrances on the part of the ratepayers, some or many of the Borough Councils within the Metropolitan area are callous as to the health conditions of the districts for which they are responsible. Londoners should therefore welcome a paper on "The Collection and Disposal of House Refuse," by Mr. C. H. Cooper, M.I.C.E., and a discussion thereafter, which has been arranged by the Royal Sanitary Institute, at 90 Buckingham Palace Road, at 5 p.m. on May 8. We are glad to note that the London County Council Public Health Committee, in dealing with the case of St. Pancras, after admitting its Council's considerable difficulty in disposing of refuse, stated that in this matter the position of St. Pancras was without parallel in any part of the country. The Health Committee therefore advised the London County Council to make representations to the Local Government Board, under Section 101 of the Public Health (London) Act, 1891, that the sanitary authorities made default in executing the provisions of that Act. Had the London County Council made themselves master of the actual state of affairs in every part of the Borough of Paddington it is highly probable that they might have found themselves justified in including that Borough, with St. Pancras, in their indictment. The truth is that residential portions of the Metropolis are suffering from the inefficiency of the administration of Borough Councils, to the depreciation of property and with increased risks to the health of the inhabitants. It is just to say that the Borough of Kensington in this matter of the removal of refuse has set an admirable example to Borough Councils, by exhibiting the capacity to provide a well-organised system, and displaying a due regard to the health and comfort of the residential population of all classes within its jurisdiction.

### THE PRINCE OF WALES' HOSPITAL, CARDIFF, FOR LIMBLESS SAILORS AND SOLDIERS.

Considerable progress has been made with the alterations at the old Mansion House, Cardiff, and the Council hope to receive the first patients on Monday, May 7. The prolonged frost during the early part of this year considerably delayed the building operations, and some weeks will elapse before the whole hospital is complete. Owing to the great need for further hospitals of this class, however, the committee requested the matron to arrange to receive patients at the earliest possible moment, and the wards are now ready for occupation. The limb workshops are being completed and will be in working order by May 7; but the technical training workshops, staff house, and officers' quarters will not be ready for occupation for another month or two. One feature that has been carefully considered by the committee is the provision of emergency exits in case of fire, and, when completed, the provision in this respect will be ample. Lord Devonport's scale of rations is being adopted at the hospital, but it is hoped that, by arranging a sufficient variety of other provisions, no deprivation will be felt by the patients.

### GRACEFUL GIFT TO KING EDWARD VII.'s HOSPITAL, CARDIFF.

Major I. A. Gibbs, of Penarth, who has been under treatment in the wounded officers' ward of King Edward VII.'s Hospital, Cardiff, handed to the matron, Miss Montgomery Wilson, before leaving the hospital, a cheque for 1,000 guineas to endow a bed in the children's ward on behalf of his little son, John, Morel Gibbs, to be called "Jack's Cot." In a letter accompanying the cheque, Major Gibbs wrote:—"I cannot leave your hospital without expressing my grateful appreciation of my treatment here. The medical officers have been very good to me, and the sister and nurses have been most kind and considerate; in fact, nothing has been lacking; and I consider that the officers and men who are lucky enough to find themselves under this roof can consider themselves most fortunate." The King Edward VII.'s Hospital, Cardiff, has a remarkable record of endowed beds. "Jack's Cot" will make the sixty-ninth bed to be endowed in the hospital. The year 1892 saw the first endowment of this kind. No fewer than nineteen of these beds have been endowed since May 1915, three, including Major Gibbs's, in the current year.

### MORE BEQUESTS FOR GLASGOW HOSPITALS.

Glasgow hospitals, which have figured as beneficiaries in quite a number of big wills of late, will receive further bequests of a substantial character under the will of the late Mr. Thomas Wharrie, of Hampstead, and of Alford and Warnham, Sussex, and a director of the Prudential Assurance Company, who was formerly principal of the civil engineering firm of Messrs. Wharrie and Colledge, of Glasgow. Mr. Wharrie's fortune amounted to over a quarter of a million sterling, and he left £1,000 each to the Royal Infirmary, Glasgow; the Western Infirmary, Glasgow; the Victoria Infirmary, Glasgow; the East Park Home for Infirm Children; and the Higginbotham Sick Poor Nursing Association.

### ENDOWMENTS FOR THE SICK POOR IN CONVALESCENCE.

The sick poor are specially remembered in the will of a Surrey gentleman, Mr. Ernest Penrose Arnold, of "Stilemans," Godalming, who died on February 15 last. Besides other charitable bequests—including one of £1,000 to the Meath Homes, Godalming—Mr. Arnold leaves one-half of the balance of a sum of £7,000 to the Children's Sanatorium at Holt, Norfolk, or other sanatorium for the poor to be selected by his executors, and bequeaths his residence, "Stilemans," with the furniture and effects therein, to the trustees of the Schiff Home of Recovery, at Cobham, to be used in perpetuity as a home of recovery for poor persons who are suffering from wounds from accident or surgical operation; to be called the "Clara Arnold" Home of Recovery for Surgical Patients. After the payment of certain legacies, the residue of the property is to be applied for the endowment and upkeep of the Home.

### THE WIDOW'S MITE.

From Cardiganshire is reported a legacy to a hospital under most remarkable circumstances. The donor is an eccentric woman named Shani, who was formerly a pauper, and later an old-age pensioner. She was a well-known character to the Glamorganshire miners and others who have been in the habit of spending their holidays at New Quay, and she evidently took great care of the little bits of silver and the coppers which she received from the visitors. It was announced at a meeting of the Aberayron Board of Guardians the other day, that Shani had made a will leaving her little hoard of these coins, amounting to £104, to the Aberayron Hospital, and the clerk, in detailing the facts of the case, said that in the woman's house was found one thousand threepenny pieces and two drawers full of coppers.

### THE LATEST HOSPITAL HISTORY.

The Wolverhampton and Midland Counties Eye Infirmary has found its historian in Dr. Chesshire, who has prepared a sketch of its progress during the thirty-five years of its existence. Even in early days, he points out, there were those who regarded Birmingham as sufficiently near to satisfy all local needs for an ophthalmic hospital; but these councillors were overruled. In 1881 a private house in Chapel Ash was taken, and 2,100 patients were treated there in the first twelve months. The income at that time included £44 from workmen's subscriptions. At the end of three years this sum had multiplied by ten, and there was a substantial balance in hand, which justified the establishment of the hospital. Soon after, the original six beds had become ten, in 1886 a scheme for the present institution was started, and the existing hospital, with twenty-five beds, was opened in 1888. The work has continued to increase, and in 1916 the hospital possessed an endowment of £18,000, which Dr. Chesshire rightly describes as a fortunate, if not exceptional, proof of prosperity. His sketch is one the hospital may be grateful for, and we hope to have the opportunity of referring to it again.

### THE PARTITION OF AN INFIRMARY CHAPLAINCY

Difficulty in connection with the vacant office of chaplain to the workhouse and infirmary at Chepstow has been surmounted in a somewhat novel manner. The filling of the vacancy had given rise to much discussion. The new vicar, the Rev. David Hughes, sent in a formal application for the post, and the Nonconformist ministers of the town also laid claim to the appointment for one of their number, offering, moreover, to discharge the duties without payment. Failing the Guardians' acceptance of this proposal, the Rev. J. Edward Brett, a Nonconformist minister, was willing to act as a chaplain in conjunction with the vicar. Mainly through the advocacy of Archdeacon Bruce—who said that the Church owed a great debt to their Nonconformist friends and that one could not spare the other—the board decided to make a triple appointment; a resolution that the vicar be elected sole chaplain, at a salary of £50 per annum, was withdrawn. With only two dissentients it was agreed to appoint the vicar, Mr. Brett, and Father Towne (Roman Catholic) as "religious instructors" at salaries of £30, £15, and £5 respectively. The Local Government Board has now signified its approval of this arrangement as a temporary measure during the war.

### A TRENCH EXHIBITION.

The final use of the site of the old Manchester Royal Infirmary has not yet been decided on, and the debate may be resumed at any time, but meanwhile it is proposed to get the soldiers to dig trenches thereon, and to collect some trophies of the war. An exhibition of these and of the trenches will then be held, and the proceeds will be given to the Red Cross Society. The idea is one which has been successfully employed at Heaton Park, where a sum of over £5,000 was raised for the benefit of the Blinded Soldiers' and Sailors' Fund. Subject to the City Council's approval, the new plan should prove as successful as its predecessor, and seems to be the only use to which the site can be put without exciting controversy.

### THE WORK OF THE LONDON HAMPSHIRE SOCIETY.

The war, which has led to the organisation of so many things, has also affected hospital visiting, and the extent to which this has been co-ordinated can be judged from the work of the London Hampshire Society. This Society was affiliated to the Conference of the London County Societies, and a year ago a scheme of hospital visitation was started. During 1916 initial difficulties were in part removed when the Controller of Military Hospitals gave a defined right of entry into a large part of the military hospitals in London. It was then found that all hospital patients required visits. The visitors passed on the names of the respective county men to the various County Societies affiliated to the Conference, and even to those which were not. Some 160 or 180 hospitals, large and small, had to be visited, and men in the Channel Islands and, indeed, in such remote places as Ceylon, needed looking after. Therefore more visitors are required. The Society is regarded as the official visitation office in London, and even the Pensions Committees make use of the information which it collects. The constant change in the *personnel* of the patients makes the work arduous and difficult, but, in spite of all this, a good deal has been done.

### AFTER THE EXPLOSION.

We have read with sympathy and interest of the work of the East End hospitals at the time of the great explosion of January. We now learn from a report of the Charity Organisation Society how the unfortunate people affected were housed, fed, and generally cared for. Within eighteen days 1,535 persons were registered as being homeless or in need of assistance. Lack of money was not the trouble, for the Government very quickly accepted entire responsibility, financial and otherwise; the first question, and a difficult one, was to provide refuge for a large number of families and individual cases. Here it will be particularly interesting to our readers to hear of the help given by convalescent homes. Many children and mothers who were not specially tied to the neighbourhood by reason of their husbands' work were sent away to convalescent homes, care being taken to choose sick children and those suffering from shock. The London Hospital and the Church Army were especially generous in this respect, and arranged for some 250 children and mothers to go, free of charge, to the various homes to which they had access. It was very difficult to persuade some of the poor people to leave the spot, as during the confusion there was a great deal of looting, and many families were found huddling together amongst the ruins of their homes, afraid to leave for fear of losing what little remained of their possessions.

### A CAREFUL RECORD OF RED-CROSS WORK.

The work of the Red Cross in Gloucestershire from October 1915 to December 31, 1916, is described with a wealth of detail and illustrated profusely by means of maps, charts, photographic views, and reproductions of *x*-ray photographs in a report issued by the County Director, which may be obtained at a cost of one shilling from the honorary secretary, Miss C. Allen, 8 St. Aldate Square, Gloucester. We mention the last fact because this is one of those records connected with the war which will not be thrown away after perusal, but will be kept as a memorial of extensive work and practical sympathy with our sick and wounded heroes. What Gloucestershire has done is no doubt what has been done in other counties, but we doubt if all counties have been so fortunate in finding an editor with the patience, ability, and spare time possessed by the editor of the present volume. In it we learn full particulars of the work of the nineteen districts into which the county has been divided for the purposes of organisation, and of the sixty or more Voluntary Aid Detachments working in those districts. In the hospitals there are 2,300 beds at the present time, most of which since early in July have been constantly in use; but the County Director says that the more beds there are the easier is the administration of a hospital, the more economical is it both financially and in *personnel*, and the greater is its efficiency.

### CRITICISM OF THE ECCLESIASTICAL COMMISSIONERS.

Some very outspoken remarks were made at an inquest in South London the other day by Dr. Pywell, of York Road, Lambeth, who strongly condemned the "disgusting" and "neglected" hygienic conditions of Quinn's Buildings, Waterloo Road, in which all sorts of slum diseases are said to be rife. He also stated that this plague spot is owned by the Ecclesiastical Commissioners, but is in the hands of Jewish agents. If these opinions and statements are well founded, it is certainly time a public body like the Ecclesiastical Commission freed itself from such a reproach; and it is in any case odd that this property of all others should be placed in the hands of Jews. That Dr. Pywell is probably correct in his version of the disgraceful state of Quinn's Buildings is to be assumed from certain statements by the medical officer of health for Southwark. He says that on several occasions notices have been served upon the agents to put the property in repair, but that nothing has been done. We suggest that it is about time the notices were served on the Ecclesiastical Commissioners themselves, not on their Hebrew understrappers; and that failing prompt and effective action a prosecution be undertaken.

### RATIONS IN V.A.D. HOSPITALS.

The Director-General of Food Economy, Mr. Kennedy Jones, M.P., has written to the Hon. Arthur Stanley, M.P., chairman of the Central Joint V.A.D. Committee, suggesting that for ordinary cases soldiers in hospital should conform to the bread and sugar rations laid down for meals in hotels. Special cases where invalid foods are required, or the doctor in charge definitely orders extra nourishment, are excepted from this suggestion. Mr. Stanley has forwarded copies of Mr. Kennedy Jones' letter to all county directors in England, Ireland, and Wales, requesting them in turn to notify the commandants of hospitals in their counties. Although everyone must desire that sick and wounded soldiers should be properly fed, the case for conformity with the rationing regulations is very strong. Most hospital cases are men taking little or no exercise, and therefore well able to thrive on the diet scale which the rest of the community is expected to subsist upon. The paragraph about special cases appears amply to meet the needs of any men adherence by whom to the rigid scale might delay or prejudice their recovery.

### A MEDICAL MAN ON RATIONS IN HOSPITALS.

The fact that the weekly cost per bed at the Westmorland County Hospital has risen only from £1 3s. 7d. in 1915 to £1 5s. 3d in the past year, a sum, for instance, below that in 1910 (when the cost per bed was £1 6s. 3d.), led Dr. Noble to make some interesting statements in presiding at the recent annual meeting. He said that there was no such thing as rationing at the hospital. Every care to economise was taken, but it would be an entirely wrong policy to attempt to ration persons in hospitals. It was the business of the hospital to get them well and discharged as soon as possible. Children, for instance, had not the reserves to stand the strain of which adults were capable. Expenditure on dressings and cotton-wool, he added, was economical for a similar reason. We wonder whether Dr. Noble would believe that it is as ill-advised to ration hospital staffs as hospital patients? Public regulations generally break down, and the art of rationing seems to us far more the art of economy than a matter of weights and scales.

### THE M.A.B. AND THE DIET SCALE OF ITS EMPLOYEES.

By rearranging the dietary of the 4,000 employees who form the staff at its various institutions, the Metropolitan Asylums Board reports that it has in sixteen weeks saved over £4,000, while the workers have been better fed than ever into the bargain. As an experiment, a common menu was drawn up each month, and within the time stated there was effected the following large saving in the quantities of certain foodstuffs issued: Milk, saving of 3,726 gallons; eggs, 43,112; tea, 15½ cwt.; sugar, 88 cwt.; flour, 36 cwt.; meat, 411 cwt.; of a total value of £4,130. These figures are based upon a comparison with those for the four weeks immediately preceding four weeks. This economy was effected, it is stated, by the substitution of other foods, by the elimination of unnecessary issues, and by alteration in the serving of meals. It was not obtained by "cutting down" the food of the staff, except in accordance with the Food Controller's scale.

### INDIAN CHILDREN AND INFANT FOODS.

The question of shipping and the wartime limitation of imports raises some peculiar problems. Recently a letter appeared in the *Times of India* in which it was suggested that among other things the importation into Hindustan of condensed milk and infant foods should be forbidden. This suggestion at once brought forth a protest from young parents. The Indian-born English child is sometimes a fragile plant, and the milk obtainable in India is not always good. In these circumstances to keep out infant foods might prejudice the lives of English children. Doubtless the motive of the suggestion was to promote the breast-feeding of English babies born in India, a matter about which a good many English mothers in India are remiss. The excuse that supplies of cow's milk are unreliable is all the more reason for promoting breast-feeding. Still, we do agree that this particular measure is not the best way to promote breast nursing, and would inflict hardships on older babies as well as on some nurslings. It is, therefore, ill-advised, and it is to be hoped that the encouragement of maternal nursing will be attained in other ways.

### CHAIRMEN WHO "FIRE" HOSPITAL MEETINGS.

OF what use is a trail of gunpowder without a spark? No chairman, however clever, can "fire" his colleagues unless he has downright enthusiasm for his philanthropic work. What the services of a man with experience, coupled with enthusiasm, are worth to a progressive institution are simply incalculable. Think what enthusiasm means when it is found in the leader of any body of men: it means inspiration, courage, will-power, assertiveness, patience, victory! And it is our voluntary hospitals more than any centres, outside the Navy and Army, where enthusiasm is most essential to-day. Charitable organisations not only squander and waste good money when enthusiasm is wanting—they bring prolonged suffering and misery upon those who have a right to expect benefit and relief. A chairman without enthusiasm is like a fountain without water, or a tree without a leaf. There will be no need for State interference on behalf of our big voluntary hospitals so long as they have chairmen who possess enthusiasm. It is enthusiasm coupled with sagacity that we must have. The pity of it all is that sagacity and enthusiasm so seldom go hand-in-hand.

### THE CRECHE AND THE WORKING MOTHER.

WE wonder, in the interests of national health, how many of the women who have undertaken war work previously performed by men, from that of railway porter to farm hand, would be anxious at this moment to return to their previous avocation if the opportunity occurred. It is clear to thoughtful people that for most, if not all, the work which they have undertaken women are unsuited, but so long as there is a plentiful supply of women this fact can be obscured, because, for the moment, they do it well, and, to the employer, cheaply. We must, at the very moment of the child welfare campaign, be doing much to encourage infantile mortality, and weakly children, by having to take so many women away from home. The crèche is no true substitute for the breast and a decent home, and hospitals have learnt more quickly than the public that, though there must be institutions, the real remedy would be not to multiply these in every district, but so to raise the income of the family that the mother need not go out to work. When peace returns many employers will doubtless be as eager to retain the services of women as the men will be to drive them out. Though the primary motive is selfishness in each case, we must hope, for the sake of the national health, that the men will be successful. The crèche is not only a remedy, it is also a symptom of infantile mortality.

### ONE HEALTH VISITOR FOR 500 BIRTHS.

A STANDARD of health visiting has been fixed. A report of the Bedfordshire County Council Health Committee states that it has received a communication from the Local Government Board pointing out that the births to be dealt with in the County Council area number 2,000 per annum. The Local Government Board standard is one health visitor for every 500 births, and as the births in the area provided for by the County Council are much more spread out than is the case in an urban community, the Board consider that some further provision should be made. There are at present three health visitors, and these, according to the Local Government Board standard, are competent to deal with 1,500 births. There remain, therefore, 500 births calling for the services of another health visitor. The Committee are informed that the Local Government Board are well satisfied with the work which has been done by the three health visitors, but they think the time has arrived when further provision should be made. This question has been carefully considered by the County Medical Officer of Health, who has suggested two alternative schemes—namely, (1) to appoint another whole-time health visitor and to give her one-quarter of the area at present served by the three health visitors, or (2) to appoint three part-time assistant health visitors out of the ranks of those practising midwifery within the county, one of such to assist each of the three health visitors. The Committee have given their most careful consideration to these suggestions, and they are unanimously of opinion that the only satisfactory course is to appoint a fourth health visitor.

### THE CASE FOR MOTHERS' PENSIONS.

AN interesting conference has been held in Birmingham on the subject of Mothers' Pensions. Many organisations were represented, and Mr. F. W. Rudland, president of the Birmingham Trades Council, was in the chair. He reminded the conference that the founder of the scheme for mothers' pensions, which has been adopted in twenty-seven of the United States, is Judge Neil, of Chicago. Its object was to provide mothers too poor to bring up their children properly with money, so that the children should not be taken away from their homes on the mere ground of poverty. Mr. George Lansbury said that the State of Illinois was the first to pass the law which provided such pensions, and a similar scheme has been adopted in Glasgow. The Town Councils, Mr. Lansbury said, should administer these pensions through the public health authorities, and the pensions should bring no taint of pauperism with them. The only condition should be that the mothers should allow a visitor now and then to see how the children were being looked after. In support of a resolution urging the introduction of such a scheme, Mrs. Barrow Cadbury said that no woman with children at home ought to go out to work.

### AN ATTENDANT EVIL OF SOCIAL REFORM.

THE principle underlying the case for mothers' pensions set out in the preceding paragraph is very important, and a timely reminder of the chief attendant evil on all social reforms. When a benevolent and enthusiastic person sees children ill or hungry, and poor people in need of this or that which they cannot provide for themselves, he hastens to raise a fund and equip an institution to provide it. Many of these institutions, like hospitals, for instance, are a necessity, since no private person, however wealthy, can afford, or even desire, the special equipment which they have. But many others, like "Homes for Children," crèches, and the like, are merely substitutes for real homes and private nurseries. They cannot, of course, be provided without rules and regulations, and, like the crèche, they maintain the very evil, that of mothers being out at work, which they were designed to palliate. Indeed, they are part of the evil; and the home is still broken up with their aid. The regulations imposed on those who benefit by these institutions may be necessary, but are irksome, and a good alternative would be to provide the families subject to them with an income, under adequate safeguards through the municipalities, which will make each of them self-respecting and independent.

### A MUNICIPAL HOSPITAL.

Having regard to the fact that the isolation hospital will shortly be receiving maternity cases and cases of general diseases among children, the Willesden Urban District Council has come to the conclusion that the institution has ceased to be an isolation hospital and decided in future to call it the Willesden Municipal Hospital. In connection with the food regulations, the Council has left it to the discretion of the medical officer to feed patients as he deems necessary.

### ROWDYISM AT SOLDIERS' CONCERTS.

Once again comes an instance of the bad behaviour of soldier patients at concert parties. This time the incident is recorded in the April number of the *Southern Cross*, the gazette of the 1st Southern General Hospital, Birmingham. A correspondent, whose protest is endorsed in the editorial, implies that interruptions at the concerts are frequent, and declares that "a certain party that occupied the gallery at the right of the stage seemed to have made up their minds to be as rowdy as possible; also a sprinkling of Colonials were evidently doing their best to get their comrades a bad name by their rude behaviour." The writer concludes by asking the soldiers "to give the artistes a fair chance, then if they do not find the performance quite to their liking, let them return to the wards they belong to." A rule to enforce this course should exist. Such bad manners is black ingratitude when performers come often at personal inconvenience to entertain the wounded, and, in addition, it helps to maintain a level of vulgarity and silliness since the word goes round that nothing more worthy is appreciated. We hope that the Commanding Officer will do his best to protect the performers in the future by excluding the men who are ill-behaved from the concerts.

### THE APRIL "SEARCHLIGHT."

Unlike most gazettes, the magazine of the 2nd Western General Hospital, Manchester, is mostly comprised of mere sketches of more or less humour, in the midst of which come as a welcome relief the articles on literature, nature, and the poets, to which one page a month is devoted. Since to soldiers in hospital the subject of the war is often one which they wish to be spared, presumably incidents of hospital life also have to them a certain monotony. We therefore believe that gazettes like the *Craigleith Hospital Chronicle*, which aim at a high standard of excellence and publish articles with a wide range of interest, are well advised. Humour, like all good things, is better in small doses, and the pseudo-comic or merely silly is the most wearisome thing in the world.

### PHARMACISTS HELP THE GREAT NORTHERN HOSPITAL.

Mr. Herbert Skinner, pharmacist of the "Great Northern," has indirectly been the means of his institution receiving a contribution of £50. It appears that the North London Pharmacists desired to give Mr. Skinner (who acts as the honorary secretary of their Association) some recognition of all his good work on their behalf. After consultation with Mr. Skinner, it was decided to arrange a social evening, all the proceeds to be handed over to the institution at which he is pharmacist. Donations, added to the takings at this "social," resulted in the sum mentioned above being handed over to the treasurer of the hospital. We congratulate Mr. Skinner on this fine testimonial, and the North London pharmacists on the method they adopted of giving it.

### THIS WEEK'S DRUG MARKET.

The main event of the week has been the notification made by the Army Council, under the Defence of the Realm Regulations, of their intention to take possession of stocks of quinine, phenacetin, and formaldehyde. Firms holding stocks of these drugs above specified quantities are required to furnish the Director of Army Contracts with particulars of their stocks. In the meantime business in these drugs is suspended. Presumably, hospital authorities whose stocks exceed 100 ounces of quinine sulphate, 7 lb. of phenacetin, or 10 gallons of formaldehyde solution (40 per cent.) will make the returns. On the whole, business in the drug market has been quiet, with few price changes of particular note. Henbane leaf is practically unobtainable, and there is very little green extract available. Opium still tends upwards in price. Makers of codeine have advanced their prices, as also have makers of diacetyl morphine and morphine ethyl hydrochloride; but there is no change in the quotation for morphine hydrochloride. Paraldehyde is rather dearer. Oil of cloves has again been advanced in price. Thymol is rather dearer. Reverting to the official notification with regard to quinine, phenacetin, and formaldehyde, it may be noted that for some time past a steady business has been passing in quinine, and of late there had been apparently little speculation in the drug. As regards phenacetin it is understood that British makers are steadily increasing their output, but this is still far from sufficient to satisfy the large demand. Formaldehyde is required in large quantities, and supplies have not been coming in in sufficient quantities.

